# The Emerging Role of Bone-Derived Hormones in Diabetes Mellitus and Diabetic Kidney Disease

**DOI:** 10.3389/fendo.2022.938830

**Published:** 2022-07-11

**Authors:** Yixuan Li, Zuhua Gu, Jun Wang, Yangang Wang, Xian Chen, Bingzi Dong

**Affiliations:** ^1^ Department of Endocrinology, The Affiliated Hospital of Qingdao University, Qingdao, China; ^2^ Department of Endocrinology and Nephropathy, Weihai Hospital, Weihai, China; ^3^ Department of Clinical Laboratory, The Affiliated Hospital of Qingdao University, Qingdao, China

**Keywords:** bone-derived hormone, fibroblast growth factor 23 (FGF23), osteocalcin (OCN), sclerostin, diabetes mellitus (DM), diabetes kidney disease (DKD)

## Abstract

Diabetic kidney disease (DKD) causes the greatest proportion of end-stage renal disease (ESRD)–related mortality and has become a high concern in patients with diabetes mellitus (DM). Bone is considered an endocrine organ, playing an emerging role in regulating glucose and energy metabolism. Accumulating research has proven that bone-derived hormones are involved in glucose metabolism and the pathogenesis of DM complications, especially DKD. Furthermore, these hormones are considered to be promising predictors and prospective treatment targets for DM and DKD. In this review, we focused on bone-derived hormones, including fibroblast growth factor 23, osteocalcin, sclerostin, and lipocalin 2, and summarized their role in regulating glucose metabolism and DKD.

## Introduction

The incidence and prevalence of diabetes mellitus (DM) are rapidly growing worldwide (1). With advances in medical therapies and the increase in life expectancy, the prevalence of DM complications is also expected to rise substantially. Diabetic kidney disease (DKD) is one of the most devastating microvascular complications of DM. DKD manifests with albuminuria regression, rapid decrease in glomerular filtration rate (GFR), or non-proteinuric or non-albuminuric DKD (2). The initiation of DKD is hyperglycemia associated, whereas the promotion of DKD is strongly related to hyperglycemia, albuminuria, hypertension, insulin resistance, and so on (3). Because of the lack of effective prevention and treatment, DKD leads to the major cause of end-stage renal disease (ESRD) and mortality in patients with DM (4). Thus, a deeper mechanistic understanding of DKD is needed.

The bone is classically considered a structural organ for supporting the human body and physical movement, safeguarding internal organs, and storing and maintaining mineral homeostasis. In recent years, bone has been established as an endocrine organ, and bone-derived hormones are involved in regulating glucose and energy metabolism (5, 6). The bone-derived hormones, as part of endocrine systems, reinforce the tight link between bone and other organs and maintain homeostasis (7).

It is conceivable that abnormalities of bone-derived hormones may lead to disorders in glucose metabolism and dysfunctions in glucose regulatory organs, such as the pancreas, liver, adipose, and kidney. From another perspective, the higher fracture risk in patients with DM also suggests that bone health and endocrine functions can be strongly affected by long-term exposure to a hyperglycemia environment (8). Among the DM complications related to bone metabolism, DKD stands out for its direct effects on mineral homeostasis (9). In this review, we focus on classical bone-derived hormones, summarize the physiological regulation of glucose metabolism, and discuss how those factors influence the DKD population. We propose that bone-derived hormones are promising therapeutic targets. In addition, in-depth studies could contribute to the prevention of DKD and improvement of patients’ quality of life.

## Bone-Derived Hormones and DKD

### Fibroblast Growth Factor 23

#### Physiological Function and Regulation of FGF23

Fibroblast growth factor 23 (FGF23), the first bone-derived hormone to be discovered, is primarily produced by osteoblasts and osteocytes (10). FGF23 mainly acts on the kidney *via* the FGF receptor (FGFR)–Klotho complex co-receptor and plays a key role in regulating calcium and phosphate homeostasis (11). In the renal distal convoluted tubule, FGF23 reduces renal calcium excretion by upregulating the expression of calcium-selective channel protein TRPV5 (transient receptor potential vanilloid channel subfamily member 5) (12). In the renal proximal tubules, FGF23 induces phosphaturia by inhibiting the expression of type IIa and IIc sodium–phosphate cotransporters (Na-Pi IIa/IIc) (13). At the same time, FGF23 also suppresses the production of 1,25-dihydroxy vitamin D3 [1,25(OH)_2_D_3_] by inhibiting renal 1α-hydroxylase activity and switching from 24-hydroxylase (13). The reduction of 1,25(OH)_2_D_3_ synthesis downregulates calcium and phosphate absorption in the intestine, leading to further decreased serum levels (13). The parathyroid glands also express the FGFR-Klotho complex, and the binding of FGF23 and FGFR-Klotho complex activates the mitogen-activated protein kinase/extracellular signal-regulated kinase 1/2 signaling pathway to inhibit parathyroid hormone (PTH) synthesis and secretion (14). In addition, FGF23 suppresses the secretion of PTH by Klotho-independent calcineurin-mediated signaling pathway (15). Renin–angiotensin–aldosterone system (RAAS) plays important role in the progression of CKD. Moreover, the RAAS inhibitors show clinical evidence to slow disease progression, not only rely on the blood pressure control but also reduce proteinuria, and inhibit RAAS activity. The effect of FGF23 on the RAAS is also noteworthy. FGF23 could suppress renal angiotensin-converting enzyme 2 (ACE2) and facilitates the production of angiotensin II (Ang II), which is both prohypertensive and proinflammatory (16). However, the direct association between FGF23 and blood pressure remains unclear. Mice with X-linked hypophosphatemic rickets (XLH) characterized by high FGF23 levels do not show hypertension, suggesting that FGF23 may not affect blood pressure directly (17). Calcitriol, PTH, and dietary phosphate are the major systemic regulators of FGF23 levels (13). The high blood levels of calcitriol and PTH stimulates biosynthesis of FGF23 in bone (13, 18). Only high-phosphate diets result in FGF23 secretion, and phosphate infusion did not affect FGF23 levels in healthy humans (19, 20). It is reported that other factors, such as iron deficiency, hypoxia, chronic inflammation, adipokines, leptin, and acidosis metabolic acidosis, also affect the circulating FGF23 level (13). However, the accurate mechanism of FGF23’s local regulation *via* paracrine remains unclear (13).

#### FGF23 in Glucose Metabolism

Serum phosphate is an important mediator between FGF23 and blood glucose. In an animal study, hypophosphatemia impairs adenosine triphosphate (ATP) production in pancreatic islet cells and results in decreased insulin secretion (21). In healthy individuals, low serum phosphate levels are associated with reduced insulin resistance (IR) (22); phosphate supplementation, especially when co-ingested with glucose, can, in turn, improve insulin sensitivity (23). Moreover, serum phosphate level is disturbed in the early progression of diabetes and the phosphate deregulation adversely affects glucose metabolism (24). In Fgf23 gene-deficient mice, hypoglycemic and increased peripheral insulin sensitivity is observed, and subcutaneous glucose tolerance is improved (25). However, the effects of FGF23 on glucose metabolism in humans are less known. A clinical trial in healthy adults reported that the increased FGF23 concentrations induced by diet did not affect fasting glucose or insulin levels (26). In addition, in vitamin D–deficient patients with impaired glucose metabolism, oral glucose loading decreased the secretion of FGF23 (26). On the other hand, the association of FGF23 with IR is controversial. Wojcik et al. indicated that FGF23 contributes to insulin sensitivity and negatively correlates between FGF23 and homeostatic model assessment of IR (HOMA-IR) in adolescents with obesity (27, 28). Hanks et al. showed that FGF23 was positively associated with HOMA-IR in community-dwelling adults (29). A large cohort of 3,115 elderly subjects with diabetes demonstrated that FGF23 levels were not related to the IR (30). More investigations are needed to explain the causal association between FGF23 and glucose metabolism in humans.

#### FGF23 in DM and DKD

The fluctuations of blood FGF23 levels are complex in patients with DM. Recently, it is reported that insulin is a negative regulator of FGF23 (31). Several studies demonstrated that blood FGF23 levels are increased in patients with T2DM (32, 33). Chronic inflammatory conditions in T2DM may result in raising FGF23 levels by overruling the suppressive effect of hyperinsulinemia (34). Furthermore, leptin, advanced glycation end products (AGEs), early renal tubular dysfunction, and the application of sodium-glucose cotransporter-2 inhibitors (SGLT-2is) may also contribute to the elevated serum levels of FGF23 (24). SGLT-2is shows significant glucose-lowering and cardiovascular-renal protective effect (35). The Empagliflozin, Cardiovascular Outcomes, and Mortality in Type 2 Diabetes (EMPA-REG OUTCOME) and the CANagliflozin cardioVascular Assessment Study (CANVAS) program showed improvements in renal outcomes, and SGLT-2is are helpful in the prevention of the development and progression of DKD (35). However, randomized studies in the last several years have discovered that the treatment of Canagliflozin, Dapagliflozin, and Empagliflozin increases serum phosphate, PTH, and FGF23 (36–38). In the CANVAS trial, it was even observed that a possible association between canagliflozin and increased fracture risk (39). SGLT-2is inhibits SGLT-2 in renal proximal tubules, directly upregulate the Na-Pi reabsorption *via* Na-Pi IIa/IIc cotransporter, and then increase serum phosphate and circulating FGF23 levels. Thus, the increase in the FGF23 levels generated by SGLT2i is noticeable, as it may result in adverse diabetes outcomes including fracture and cardiovascular events. FGF23 levels started to increase early in the course of chronic kidney disease (CKD), which occurs before the increase of blood phosphate level (40). In parallel with declining kidney function and decreasing phosphate clearance, FGF23 levels will elevate further and be more than a thousand-fold higher in end-stage renal disease (ESKD), compared with normal value (41). In addition, Carlson et al. reported the intradialytic clearance of FGF23 occurs in patients undergoing chronic hemodialysis, and the clearance of FGF23 is related directly to the ultrafiltration rate (42). However, the intradialytic plasma concentrations of FGF23 remained unchanged (42). A cross-sectional study revealed that patients with DM with IR exhibited higher FGF23 levels in the CKD stages 3–5 (43). Osteoporosis is also common in the DM population. A prospective study of 126 patients with T2DM with CKD stages 2–3 suggested that patients with a fracture event displayed higher levels of FGF23, and FGF23 could independently affect the occurrence of fracture (44). On the other hand, increased serum FGF23 levels increase all-cause and cardiovascular mortality in patients with T2DM, especially under the CKD conditions (45). Some studies revealed possible mechanisms by which FGF23 affects CKD progression. In wild-type mice, elevating FGF23 levels increase hepatic and circulating cytokines and drive inflammatory states, which is associated with poor clinical outcomes of CKD (46). FGF23 signaling also impairs leukocyte recruitment *in vitro* and *in vivo* during CKD, and the disordered leukocyte recruitment increased predisposition to infections by weakening host response (47). However, in patients with CKD with hemodialysis, high FGF23 is not the cause of infection or systemic inflammation but is positively associated with vascular calcification (VC) (48, 49). VC is highly prevalent in DM and is deemed to participate in the pathogenesis of uremic VC (50). Because elevated circulating FGF23 level is associated with cardiovascular mortality and progression in CKD, the effect of phosphorus restriction diet–induced FGF23 reduction was investigated. The result suggested that a standard low-phosphorus diet reduced circulating FGF23 level in both early and advanced CKD. In addition, serum PTH was decreased in the advanced CKD group, and 1,25(OH)_2_D_3_ levels was increased in the early CKD group (51). Another randomized controlled crossover study suggested that very low–protein diet with a consequently low intake of phosphorus could rapidly reduce FGF23, serum phosphate, and urinary phosphate excretion within 1 week (52). In nephrectomized (Nx)–induced uremia rat model, serum phosphate, urinary phosphate excretion, serum FGF-23, and PTH were significantly lower in the low dietary phosphate group. Modification of phosphorus concentration in the diet affected the apoptosis of enterocytes and type IIb sodium–phosphate cotransporters (Na-Pi IIb) and phosphate inorganic transporter-1/2 (PiT-1/2) expression in jejunum mucosa (53). Those studies suggest that early control of phosphorus intake prevent FGF23 increasing and improve the VC and CKD progression. Further studies are warranted to clarify the potential role of high plasma FGF23 levels in CKD. FGF23 could be new hope for the prevention and treatment of DKD.

### Osteocalcin

#### Physiological Function and Regulation of OCN

OC is an osteoblast-secreted and vitamin K–dependent protein and is comprised of two forms: undercarboxylated osteocalcin (ucOCN) and carboxylated osteocalcin (cOCN) (54, 55). Circulating OC is accessible to measurement, and ucOCN is considered to be the bioactive form of OC that plays a role in regulating energy metabolism and glucose homeostasis (56). OCN maintains calcium homeostasis and facilitates bone mineralization and growth (55). The specific receptor of ucOCN is G protein–coupled receptor class c group 6 member A (GPRC6A), expressed broadly in various organs except for the brain. In mice models, ucOCN affects adipocyte gene expression and reduces fat mass (57). In addition, ucOCN is also required and sufficient to strengthen the exercise capacity of skeletal muscle (58). More importantly, OCN could directly stimulate β-cells proliferation, insulin secretion, and insulin sensitivity (59, 60). Mizokami et al. further found that ucOCN also induces glucagon-like peptide-1 (GLP-1) release from the gut that plays a main role in insulin secretion stimulated by ucOCN (61). Beyond that, OCN makes it possible that a more mixed regulation between bone and islet β cells. Insulin could increase OCN activity and suppress osteoprotegerin (OPG) expression that enhances bone resorption *via* osteoclasts (62). On the other hand, Delta-like 1 (DLK1) produced by pancreatic β cells could be stimulated by ucOCN and negative feedback regulate the OCN production in osteoblasts (63). In addition, ucOCN modulates reproductive function by situating testosterone secretion from the testis (64). G protein–coupled receptor 158 (GPR158) acts as the receptor for ucOCN in the brain, and through binding to which ucOCN enhances the brain’s cognitive function (65).

#### OCN in Glucose Metabolism

The above summary of OCN functions, based on the experimental models, provides preliminary evidence for the connection between OCN and glucose metabolism. The evidence of OCN that directly impacts glucose metabolism is also accumulating in humans. A cross-sectional study of 2,353 participants showed that the serum OCN level was highest in the normal glucose tolerance (NGT) group and gradually reduced in the impaired glucose tolerance (IGT) group and T2DM participants (66). After this, 1,049 participants with no diabetes and 983 participants with NGT were follow-up for 4 years, and researchers reported that the low serum OCN level group (<23.33 ng/ml) exhibited an increased risk of T2DM, impaired fasting glucose (IFG), and IR (66). A prospective community-based cohort study, which consists of 6,595 middle-aged to elderly Chinese participants, demonstrates that high circulating OCN was significantly associated with decreased blood glucose level, IR, triglyceride (TG), and body mass index (BMI) (67). Thus, OCN may correlate positively with glycemic metabolism status, and lower serum OCN concentration is associated with incident T2DM, which was also justified in different populations by the subsequent studies by Urano et al. and Ye et al (68, 69). However, a prospective investigation showed that there was no evidence of an association between ucOCN and incident T2DM in older participants (70). Aside from this, OCN is a medium through which some medication affects glucose metabolism. Randomized clinical implementation trials have proved that the glucocorticoid through decreasing OCN concentrations reduces hepatic insulin sensitivity and induces basal and postexercise IR (71, 72). Interestingly, the decline of OCN caused by medication is not always influencing blood glucose levels. Lewis et al. discovered glycated hemoglobin (HbA1c) did not alter clearly although OCN was fall in older women after 1 year of calcium supplementation (73). Future research may assess whether treatment with more profound effects on OCN interferes with glucose metabolism.

#### OCN in DM and DKD

Many basic experimental studies have proved OCN engages in different stages of DM development and play a protective role through influencing adipose tissue metabolism, pancreatic function, and oxidative stress (74). In a clinical trial involving 75 middle-aged to aged Japanese without any anti-diabetic agent administration, it was observed that ucOCN is correlated with HbA1c and insulinogenic index (IGI) in the DM group, and ucOCN plays more vital roles in insulin secretion than in insulin sensitivity in patients with DM (75). In a study of children with newly diagnosed DM, serum C-peptide levels are related to a higher ucOCN and ucOCN/cOCN ratio (76). The abovementioned research suggested OCN favors insulin secretion in patients with DM, but the relationship between OCN and glucose homeostasis, which is crucial for controlling the progression of DM complications, is uncertain. A previous study investigated the community-based adults with type 1 DM (T1DM), finding that OCN is unrelated to any glucose homeostasis marker (77). Nevertheless, the relationship between OCN and DKD has been well documented. A 4.6-year prospective study of 1,174 patients with DM with normal kidney function concluded that lower OCN levels were relevant to an increased risk of incident DKD (69). A cross-sectional study induced 374 men and 364 postmenopausal women showed that patients with T2DM with micro or macro-albuminuria had lower OCN levels compared with patients with normal albuminuria (78). In addition, the decreased OCN levels could affect osteogenesis in T2DM with proteinuria (78). Similar to FGF23, OCN also independently affected the occurrence of bone fracture in patients with DKD and was lower in patients with a fracture event compared with patients without fructure (44). Hemodialysis is an important therapeutic choice for patients with ESKD. Carlson et al. observed that OCN blood concentrations declined during hemodialysis but rebounded within 6 h, and the intradialytic plasma concentrations of OCN did not change significantly (42). Fusaro et al. found that patients with DM undergoing hemodialysis had a higher risk of all-cause mortality and total OCN and ucOCN were lower, compared with patients without DM undergoing hemodialysis, which might indicate that OCN plays a potential protective role in patients with ESKD (79). A lower OCN level is unfavorable for blockading the onset and progression of DKD. Given that ucOCN is the active form of OCN, more clinical research studies are necessary to evaluate the role of ucOCN in the DKD population.

### Sclerostin

#### Physiological Function and Regulation of Sclerostin

Sclerostin is a glycoprotein predominantly produced by mature osteocytes, and it can inhibit bone formation by occupying Wnt coreceptors low-density lipoprotein receptor-related proteins 5 (Lrp5) and Lrp6 to suppress Wnt signaling pathway (80). The latest study supplemented that the binding of sclerostin to Lrp4 enhances this suppression by facilitating sclerostin-Lrp6 binding (81). The changes in Wnt signaling also stimulate bone resorption by repressing the expression of OPG, a downstream target of the Wnt signaling pathway that can inhibit bone resorption (82). Therefore, sclerostin effectively reduces bone mass and volume, and antisclerostin monoclonal antibody has gradually held an important place in the treatment of osteoporosis (83). In addition, sclerostin can affect mineral metabolism by altering mineral homeostasis-related hormones. Sost is the gene encoding sclerostin, and Sost^−/−^ mice display lower FGF23 levels, reduced calcium excretion, and elevated serum phosphorus (84). On the other hand, the Wnt pathway is also been linked to adipogenesis (85). Sost^−/−^ mice exhibit a notable increase in bone formation and a decrease in visceral and subcutaneous adipose, which are explained by the sclerostin deficiency, blocking the differentiation of adipocyte progenitors to mature adipocytes (86). In addition to white adipose tissue, the major component of visceral and subcutaneous adipose, sclerostin, also increased the brown adipose tissue (BAT)–specific gene expression and the bone marrow adipose tissue (BMAT) formation that were confirmed in other experiments (87, 88). Thus, inhibiting sclerostin also contributes to the treatment of obesity. In addition, it has been proposed that sclerostin’s regulation of adipogenesis also affects the immune cell maintenance, and sclerostin depletion is disadvantageous for B lymphopoiesis and myelopoiesis, even hematopoiesis (89). Mechanical force can promote bone formation, and the Wnt signaling pathway represents a critical role in the regulation of mechanical stress-induced bone formation (90). Hence, sclerostin then becomes an obligatory step for this process and can be downregulated by mechanical force to increase bone formation (90). Furthermore, Sost transcription is negatively regulated by PTH, and Lrp4 plays an integral role in this process (91).

#### Sclerostin in Glucose Metabolism

As stated above, sclerostin promotes adipogenesis. Adipose tissue has endocrine function and exerts an impact on energy metabolism. Numerous studies showed that sclerostin could influence glucose metabolism. Lrp4 is necessary for normal sclerostin function and is expressed in both adipocyte and osteoblast (92). Kim et al. found that mice with Lrp4-deficient adipocytes showed increased glucose and insulin tolerance and that mice with Lrp4-deficient osteoblasts developed impairments in glucose tolerance and insulin sensitivity (92). Following this experiment, Kim et al. conducted another study in Sost^−/−^ mice and observed improvements in glucose metabolism (86). Similar research was also performed on children and adolescents. Wedrychowicz et al. identified that sclerostin correlated negatively with HOMA-IR, and this correlation was stronger in obese children and adolescents (93). These investigators also found an inverse association between sclerostin and insulin in the obese group and an inverse association between sclerostin and C-peptide in the health cohort (93). These results are complemented by a recent study that sclerostin was also inversely related to fasting glucose in obese children and adolescents, and the negative relationship between sclerostin and fasting insulin levels has been also observed (94). Consequently, sclerostin is closely correlated to blood glucose level and insulin resistance. In addition, to better understand how sclerostin affects glucose metabolism in the human body, further in-depth research focusing on the potential mechanism is required.

#### Sclerostin in DM and DKD

In an *in vitro* experiment, investigators found high glucose (HG) and AGEs significantly increased sclerostin expression in osteocytes, and this function can be antagonized by PTH (95). The increased expression of sclerostin is also observed in streptozotocin-induced DM rats, which further confirmed the detrimental effects of sclerostin on bone in patients with DM (96). A case-control study including 40 T1DM cases and 28 healthy controls showed that serum sclerostin levels were negatively associated with HbA1c in patients with T1DM and the sclerostin levels were significantly greater compared with healthy participants (97). Another clinical study involving T2DM postmenopausal women found that T2DM upregulates the expression of Sost and AGEs, contributing to the impairment of bone microarchitecture (98). A prospective cohort that included 1,778 individuals revealed no clear association between sclerostin and T2DM risk (99). However, in the cross-sectional study, Napoli et al. and Shalash et al. suggested that serum sclerostin levels in patients with T2DM were noticeably higher than those subjects without DM (100, 101). In addition, the positive correlation between sclerostin and VC, sclerostin and atherosclerosis, and sclerostin and arterial stiffness in patients with T2DM was well-proved by cross-sectional studies (101–103). In addition, a protective role of sclerostin in VC development was demonstrated in Sost^−/−^ mice (104). Moreover, Jean et al. found higher sclerostin levels are associated with a better survival rate in patients undergoing hemodialysis (105). Like the two mentioned hormones, the concentrations of sclerostin also remained nearly constant, although it can be cleared during hemodialysis (42). In patients with DKD, Kim et al. detected the sclerostin level begin to elevate in CKD stage 3 and dramatically elevate in CKD stage 4/5 (106). In addition, Wu et al. found that urinary sclerostin is positively related to fractional excretion of magnesium in patients with DKD or patients with T2DM without CKD (107). The above results implied that the increased sclerostin level is probably a protective phenomenon and that urinary sclerostin also plays a potential role in renal electrolyte excretion in patients with DKD. Additional research is warranted to shed light on this phenomenon.

### Lipocalin-2 (LCN2)

#### Physiological Function and Regulation of LCN2

LCN2 is a 198–amino acid adipocytokine, also termed neutrophil gelatinase–associated lipocalin (NGAL) (108). It exists in a wide variety of cells, such as neutrophils, hepatocytes, adipose tissue, renal cells, and bone marrow (108). Megalin/glycoprotein (gp) 330 and solute carrier family 22 member 17 (SLC22A17) or 24p3 cell-surface receptor (24p3R) are two receptors that bind human LCN2 and LCN2 mouse protein, respectively (108). LCN2 plays an essential role in normal bone formation and participates in the endocrine function of the bone (109, 110). Some experiments in mice have demonstrated that osteoblast-secreted LCN2 can promote adaptive β-cell proliferation, induce insulin secretion, improve insulin sensitivity, and inhibit food intake (110, 111). The melanocortin 4 receptor (MC4R) is a key receptor for controlling food intake (110). LCN2 can activate the MC4R-dependent anorexigenic (appetite-suppressing) pathway, decreasing fat mass and body weight (110). In addition, LCN2 has bacteriostatic properties that make it competent for combating infection, injury, and other cellular stress (108, 112). LCN2 also plays an important role in cell differentiation, apoptosis, cancer progression, and metastasis (108). Pathologic conditions such as inflammation and metabolic diseases can upregulate the expression of LCN2, and LCN2 can be found in the brain, heart, and skeletal muscle that do not express LCN2 under normal conditions (108, 113–115). Moreover, AGEs, insulin, and dexamethasone are strong facilitators of LCN2 expression and secretion (108).

#### LCN2 in Glucose Metabolism

LCN2 has been shown an intimate association with the metabolism of glucose. Capehorn et al. confirmed that improved insulin sensitivity and suppressed gluconeogenesis were present in** **LCN2 knockout (LCN2KO) mice (116). Currò et al. also reported that LCN2 was positively correlated to the homeostatic model assessment (HOMA) index in normal subjects, which suggests a regulatory role of LCN2 in IR (117). Furthermore, Capulli et al. observed that LCN2KO mice showed lower fasting glucose and higher glucose tolerance compared with wild-type mice (109). These investigators also found that insulin levels were increased and the insulin tolerance remained mostly unchanged in LCN2KO mice (109). In addition to this, Mosialou et al. considered that the elevated circulating LCN2 levels are a protective reaction to resist obesity-induced glucose intolerance (110). In addition, a cross-sectional study involving 2,519 Chinese aged 50–82 years observed that the serum LCN2 was remarkably higher in subjects with IFG and/or IGT and newly diagnosed T2DM than in healthy individuals (118). Another notable finding is that the circulating levels of LCN2 are related to intrapancreatic fat deposition but not to fatty liver (119). The effect of this ectopic fat deposition on glucose metabolism merits further investigation.

#### LCN2 in DM and DKD

DM, considered a circumstance of metabolic inflammation, could lead to a certain impact on plasma LCN2 concentrations. Takaya et al. reported that the levels of LCN2 were higher in adolescents with T2DM compared with the normal control group (120). Shahnawaz et al. stated that LCN2 was significantly increased in subjects with T2DM with chronic hepatitis B infection (121). The findings from Huang et al. suggested that the elevated serum LCN2 is independently correlated with T2DM in middle-aged and elderly Chinese patients (118). A 5-year prospective study in postmenopausal women with prediabetes found that there was a strong positive association between circulating LCN2 levels and insulin levels, HOMA-IR, homeostatic model assessment of β-cell function (HOMA-B), and BMI (110). However, De la Chesnaye et al. and Wang et al. observed that the levels of LCN2 were decreased in individuals with long-term T2DM and inversely related to HbA1c and BW in diabetes (122, 123). From this, the actual changes in LCN2 levels are more complicated than expected. Interestingly, the relationship between LCN2 and the neuropathy of diabetes is well demonstrated. In mouse models of DM, LCN2 plays a critical role in the pathogenesis of diabetic encephalopathy (124). In addition, a recent study of subjects with T2DM revealed the role of LCN2 in diabetic peripheral neuropathy (DPN) and highlighted the value of LCN2 in the evaluation of DPN severity (125). Otherwise, LCN2 is also identified as the biomarker for acute and chronic kidney injury (108). Capulli et al. found that LCN2KO mice exhibited polyuria, glycosuria, proteinuria, and renal cortex vacuolization (109). Li et al. reported that the variants of LCN2 in human urine were correlated with renal dysfunction (126). In the kidneys of obese prediabetic rats, the elevated LCN2 expression occurred earlier than the biomarkers of inflammation, oxidative stress, and fibrosis, which means that LCN2 is an important predictor of early kidney injury (127). Whether the clearance of LCN2 is affected in DKD is not clear, and more comprehensive studies determining the role of LCN2 in human DKD are urgently needed.

## Future Prospects

**Figure 1:**
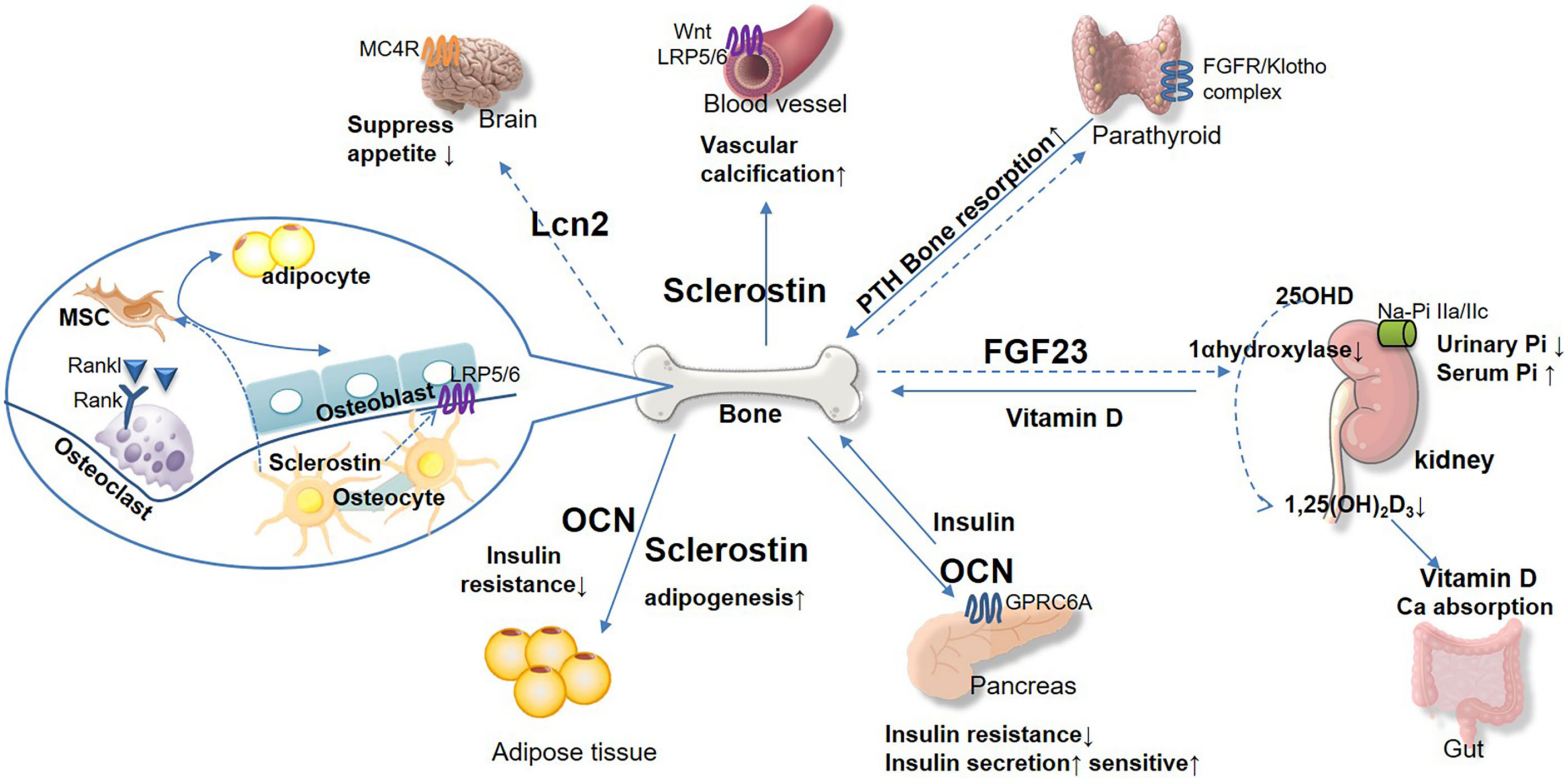
The emerging role of bone-derived hormones in glucose metabolism and cross-talk in kidney, pancreas, and other diabetic complication target organs. Bone acts as the endocrine organand links kidney and glucose metabolism. Bone-derived hormone FGF23 is secreted from osteocytes and regulates urinary phosphorus excretion from kidney. FGF23 suppresses 1 α-hydroxylase activity, inhibits 1,25(OH)2D3, and reduces parathyroid hormone (PTH) level. Osteocalcin (OCN) acts on pancreas and adipose tissue to regulate glucose and energy metabolism, insulin secretion, and insulin resistance. Sclerostin plays important role in vascular calcification, which promotes chronic kidney disease progression directly and indirectly. Osteoblast secreted lipocalin-2 (LCN2) regulates central food intake and pancreatic β-cell proliferation, to maintain glucose and energy homeostasis. The bone-derived hormones would be potential therapeutic target for DM and complications based on their effect on maintaining glucose homeostasis and bone health.

With the aid of bone-derived hormones, an intimate relation between bone and glucose metabolism has been noticed (**Figure 1**). Bone-derived hormones play an emerging role in the treatment, prevention, and prediction of DM. In addition, we summarized the relevant therapeutic studies around the bone-derived hormones in DM and DKD.

The description of FGF23 hinting to reduce its serum level is a reliable treatment option. The reduction of FGF23 levels was observed in the studies on pharmacological treatments (for example, dapagliflozin) of DKD (37). On the other hand, anemia is common in patients with CKD, and targeted therapy against it, for example, erythropoietin (EPO)–stimulating agent treatment, can induce increased FGF23 serum levels (128). Thus, more therapy strategies for patients with DKD should be considered associated with FGF23. Burosumab, the monoclonal antibody that targets and blocks the activity of FGF23, has been studied comprehensively in the treatment of mineral disorder and is expected to provide a potential choice for improving mineral metabolism in patients with DKD (129, 130). It leads an important future direction that evaluating the efficacy and safety of the anti-FGF23 monoclonal antibody in patients with DKD, which is beneficial in reducing the risk of fracture and lowering the incidence of adverse cardiovascular events. In addition, deity phosphorus restriction in patients with CKD reduces circulating FGF23 level and then improves the VC, cardiovascular outcomes, bone metabolism, and disease progression. Previous studies of mice showed that OCN appears to be a viable therapeutic method in obesity and insulin resistance. A recent study provided further evidence that the short- and long-term treatment of decarboxylated OCN (dcOCN), a kind of uncarboxylated OCN, can increase glucose uptake in MG63 cells (human osteoblast-like osteosarcoma cells), which implies that dcOCN may be a potential approach for T2DM (131). Otherwise, the clinical application for OCN as a predictor of DM complications is also underway. Zhu et al. found that circulating OCN can emerge as a predictor of ketosis in T2DM (132). In recent years, the role of OCN in gestational DM (GDM) is also arousing attention. Song et al. demonstrated that the synthesis of OCN can occur in the placenta and that a lower OCN concentration in umbilical vein serum is related to GDM (133). Inhibition of sclerostin is an effective way to lower T2DM-associated fracture risk. However, a meta-analysis indicated this therapeutic approach may lead to increased cardiovascular events (134). Thus, it calls for utmost vigilance that the cardiovascular safety of the application of sclerostin inhibitors in patients with DM and DKD. LCN2, as a novel bone-derived hormone, plays an active role in energy metabolism. The administration of exogenous LCN2 can reduce food intake and fat mass (110). On the other hand, it is also noticeable that LCN2 is a promising diagnostic biomarker and drug target in neuropathy of diabetes (135). The small-molecule LCN2 inhibitors and neutralizing antibodies against LCN2 are important future directions for the treatment of diabetic neuropathy (135). Moreover, the activation of epidermal growth factor receptor (EGFR) and the expression of LCN2 are often found in the same pathologic conditions, such as CKD (136). In addition, in the CKD model, the inactivation of the LCN2 gene can prevent EGFR recycling to the plasma membrane, which is related to a dramatic reduction of renal lesions (136). Thus, the therapeutic suppression of LCN2 may be useful to counteract kidney damage.

In short, studies on bone-derived hormones have just begun, and large prospective studies are still necessary to infer more causal relationships. In future work, more novel agents for the treatment of DM will emerge by focusing on the endocrine function of bone.

## Conclusion

Bone has long been known for its supportive and protective function. However, the endocrine function of bone deserves more attention in recent DM studies. Bone-derived hormones correlate with insulin secretion, insulin resistance, and glucose metabolism and are implicated in the development and outcomes of DM and DKD. Bone-derived hormones would be promising therapeutic targets for DM and complications based on their potential effectiveness in maintaining glucose homeostasis and bone health.

## Author Contributions

YL wrote the manuscript. ZG, JW, and YW provided helpful suggestions. XC conceived and designed the study. BD designed this study, created and prepared the figures, supervised the project, and takes responsibility for this study. All authors have read and agreed to the published version of the manuscript.

## Funding

This work was supported by a grant from the National Natural Science Foundation of China (Grant No. 81600691) and was China Postdoctoral Science Foundation funded project (Grant No.2018M640615). The content of the article has not been influenced by the funders.

## Conflict of Interest

The authors declare that the research was conducted in the absence of any commercial or financial relationships that could be construed as a potential conflict of interest.

## Publisher’s Note

All claims expressed in this article are solely those of the authors and do not necessarily represent those of their affiliated organizations, or those of the publisher, the editors and the reviewers. Any product that may be evaluated in this article, or claim that may be made by its manufacturer, is not guaranteed or endorsed by the publisher.

## Abbreviations

DKD, diabetes kidney disease; ESRD, end-stage renal disease; DM, diabetes mellitus; FGF23, fibroblast growth factor 23; OCN, osteocalcin; LCN2, lipocalin-2.
